# Editorial: Case Reports in Visceral Surgery

**DOI:** 10.3389/fsurg.2022.845298

**Published:** 2022-01-28

**Authors:** Andrew A. Gumbs, S. Vincent Grasso, Milton A. Gumbs

**Affiliations:** ^1^Département de Chirurgie Digestive, Centre Hospitalier Intercommunal de Poissy/Saint-Germain-en-Laye, Poissy, France; ^2^Family Christian Health Center, Harvey, IL, United States; ^3^Bronx-Lebaon Hospital Center, Bronx, NY, United States

**Keywords:** visceral surgery, case report, pancreatic surgery, laparoscopy—“open procedure, ” carcinomatosis

After serving for several years as a review editor for Frontiers in Surgery, I was asked last year to become an Associate Editor of Frontiers in Surgery. A few months earlier I agreed to become the Editor-in-Chief of a new journal called Artificial Intelligence Surgery (AIS), the first journal to focus exclusively on artificial intelligence (AI) and surgery (1). I was impressed by the level of organization of Frontiers in Surgery and I have used what I have learned at Frontiers to help improve AIS, and thanks to these insights our first issue (www.aisjournal.net) was successfully launched in December 2021. I was asked to contribute an Editorial to a collection of Case Reports, which I agreed to do because my love of writing about surgery all began after I wrote my first Case Report. Unlike case series and review articles, Case Reports are much more intimate accounts of our interactions with patients and in many ways more poignant.

People not in the medical field will often ask me why I write articles and publish them in journals free of charge. Doctor means “teacher,” and any academic surgeon knows that if you really want to understand a problem, the best thing to do is to write about it. I will always remember my first Case Report, a patient underwent a duodenopancreatectomy for a mass in both the gallbladder and in the head of the pancreas (2). I was so surprised to find out that the patient had autoimmune pancreatitis and could possibly have avoided an unnecessary major abdominal surgery with a simple blood test that I felt obliged to broadcast this case in an effort to help surgeons avoid this in the future. A case report discussing the pre-operative misdiagnosis of a dilated cystic duct (Zhang et al.) reminded me of this lesson.

Almost 20 years ago when I was doing a Pancreatic Surgery Fellowship in Verona Italy, I heard about a patient with a glucagonoma and migratory necrotizing dermatitis and felt compelled to ask the patient permission to take anonymized photographs of this rare rash because I had never seen it and wanted as many people to learn from this rare case as possible (3). This is why I chose this Case Report of an equally rare pancreatic tumor (Guo et al.) for this collection.

As a young Attending I found myself knee deep in an extremely hostile abdomen only to learn upon reading the final pathology that the patient had primary sclerosing peritonitis (4). This case reminded me that extremely rare pathologies can present with carcinomatosis and that essentially no form of carcnomatosis acts in a benign manner. A theme reiterated in this Case Report of primary peritoneal carcinosarcoma (Erz et al.).

I remember calling my old boss to the operating room because I could not believe that the patient could have an annular pancreas wrapped around his duodenum that was not seen pre-operatively. This patient was undergoing a laparoscopic distal pancreatectomy for a premalignant pancreatic cyst in the tail of the pancreas and had undergone every preoperative imaging exam of the pancreas imaginable (5). The innumerable diagnoses that can occur around the duodenal area is reinforced by this article of a paraganglioma that was initially believed to be a duodenal gastrointestinal stromal tumor (Zhang et al.).

Case Reports can herald the previously considered unthinkable (6), and they can be cathartic. I had a young man urgently transferred to my care in New York City from across the country, only to succumb to his disease the day after I saw him even though his minimally invasive splenectomy had been scheduled for the very next day (7). He was treated for inflammatory bowel disease with an immunosuppressant that is now known to increase the risk for splenic lymphoma. I like to think that it was some comfort to his family that his loss was not in vain, as hopefully his case acted as a cautionary tale for future doctors.

Although Pubmed has been revolutionary in the fact that surgeons can now search and find published evidence to help them with rare cases or complex issues, the process is still cumbersome and time consuming. It is hoped that machine learning (ML) algorithms of artificial intelligence (AI) can 1 day accelerate this transmission of data. Electronic access to surgical textbooks is also an innovation that deserved mention, but due to the rapidity that new data is being published and the sheer volume of literature today still highlights the need for artificially intelligence platforms to synthesize and enhance the transmission of the latest information in a clear, concise and rapid way.

Lastly, for surgeons with family members in the field, Case Reports can bring give you insights into your family's own experience into the wonders of surgery (8), and remind you how you benefitted from the ability to stand on the shoulder of giants and push the envelope further (9). Reading this article discussing a Bochdalek hernia complicated by a bowel perforation into the chest (Ramspott et al.) reminded me of the above-mentioned article that my Father authored and showed me decades ago in an effort to get me interested in academic surgery. This article more than perhaps any of the ones in this collection inspired me to agree to edit this collection. In short, Case Reports are where the journey of academic surgery truly begins and act as the gateway for us all to fall in love with the art of surgery.

## Author Contributions

All authors listed have made a substantial, direct, and intellectual contribution to the work and approved it for publication.

## Conflict of Interest

The authors declare that the research was conducted in the absence of any commercial or financial relationships that could be construed as a potential conflict of interest.

## Publisher's Note

All claims expressed in this article are solely those of the authors and do not necessarily represent those of their affiliated organizations, or those of the publisher, the editors and the reviewers. Any product that may be evaluated in this article, or claim that may be made by its manufacturer, is not guaranteed or endorsed by the publisher.
